# A Non-Fatal Impalement Injury to the Right Thorax: A Case Report

**DOI:** 10.7759/cureus.33517

**Published:** 2023-01-08

**Authors:** Adam J Mann, Lawrence Lottenberg, Faris Azar, Nir Hus

**Affiliations:** 1 General Surgery, Florida Atlantic University, Boca Raton, USA; 2 Surgery, Florida Atlantic University/St. Mary's Medical Center, West Palm Beach, USA; 3 Trauma, Florida Atlantic University/St. Mary's Medical Center, West Palm Beach, USA; 4 Trauma, Delray Medical Center, Delray Beach, USA

**Keywords:** impalement, thoracic surgeries, general thoracic surgery, vascular exposure, extremity vascular trauma, complex trauma, major trauma

## Abstract

Impalement injuries secondary to motor vehicle collisions are rare. Reviewing the systematic approach to treating these injuries can benefit the surgical community. This case report discusses the presentation and management of a 32-year-old male who was involved in a high-speed, roll-over motor vehicle collision. The car struck a chain-link fence, and the unrestrained passenger sustained a fence post impalement injury to his right thorax. He was alert and oriented when emergency services arrived. He was extricated rapidly and transported to our level-one trauma center, where he received definitive operative management. The patient survived the injury and was discharged home. This case highlights key points in the systematic approach to impalement injuries, including minimal handling of the impaled object, expedient transfer to the local trauma center, emergent operative intervention, vascular control prior to removal of the foreign object, and aggressive irrigation and debridement of the wound.

## Introduction

Motor vehicle accidents are among the leading cause of death in the United States. In 2020, the Centers for Disease Control and Prevention reported that motor vehicle collisions caused over 41,000 deaths, attributing to 2.1 million emergency department visits and medical costs of an estimated $430 billion [1]. Most of these injuries are secondary to blunt trauma; however, penetrating trauma and occasionally impalements do occur [2].

Impalement injuries are rare, which explains the paucity of these injuries reported in the literature. A review of the literature revealed 30 reported cases of thoracoabdominal impalements, only 14 of which were isolated thoracic impalements [3]. Given the rare and dramatic presentation of impalement injuries, discussing their systematic approach and perioperative management can benefit the surgical community.

We present the relevant details of a 32-year-old man who was involved in a high-speed motor vehicle accident and sustained an impalement injury through his right thorax.

## Case presentation

On a summer evening, emergency services were alerted about a four-door motor vehicle involved in a roll-over collision on a local roadway near our level-one trauma center. They arrived at the scene within four minutes to discover a vehicle that sustained heavy damage and struck a chain-link fence. Two passengers were entrapped in the two front seats. Debris and tree branches surrounding the vehicle were removed, and the passenger door was opened.

The unrestrained driver was pronounced dead at the scene. The front-seat unrestrained passenger was alert and oriented with a Glasgow Coma Scale score of 15. A 180cm metal fence post had penetrated through the windshield, impaled through his right upper chest, and exited through the passenger seat. The patient did not appear to be in extremis. There was no active hemorrhage, and initial vital signs were within normal limits including an oxygen saturation of 98% on a 4-liter nasal cannula. Extrication was prolonged because the foreign object was cut with a reciprocating saw. The injury sites were dressed with bulky gauze. The patient was placed onto a backboard in the left lateral decubitus position and transported via trauma hawk to our trauma center.

**Figure 1:**
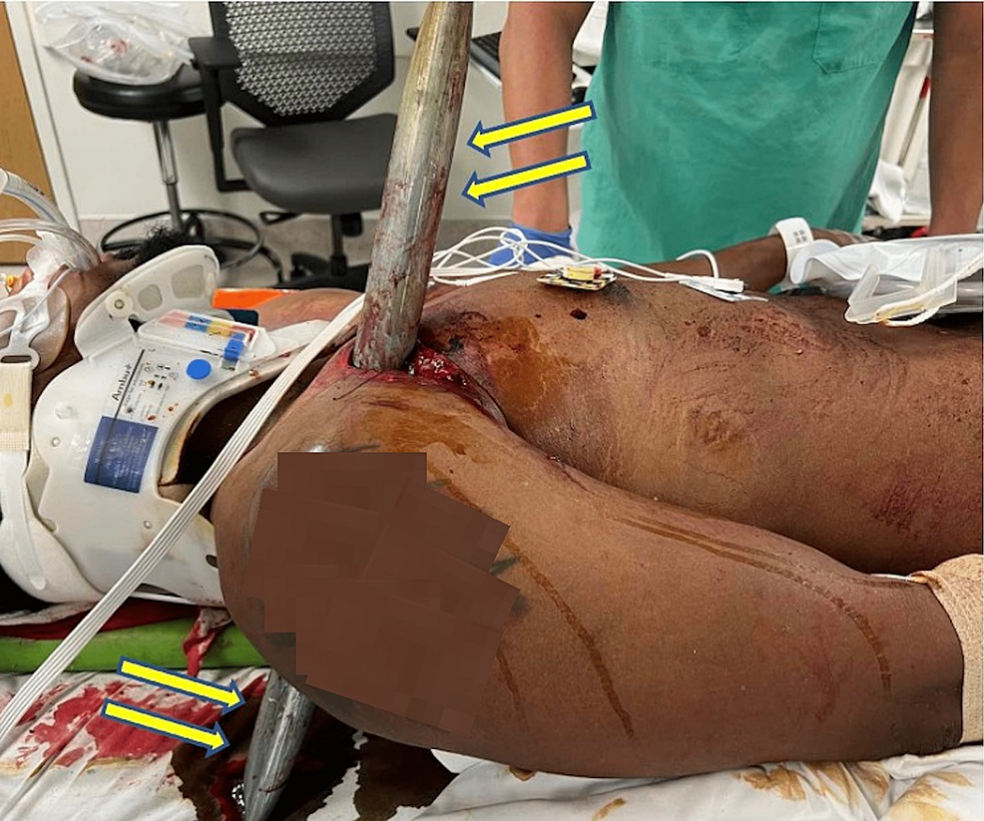
The unrestrained passenger of the vehicle is seen in the trauma bay prior to transport to the operating room. The impaled object is indicated by arrows and can be seen protruding 50cm anteriorly and 28cm posteriorly through his right upper chest wall.

**Figure 2:**
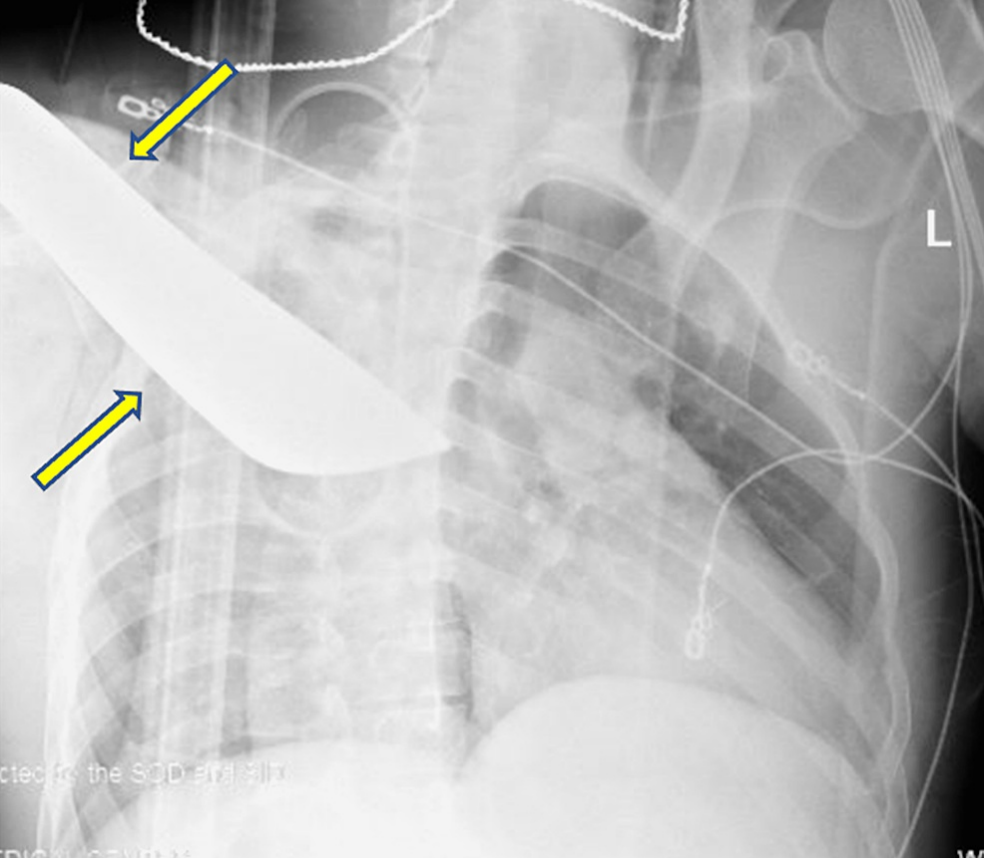
Chest X-ray taken in the trauma bay prior to surgical intervention shows the impalement object (arrows), and no obvious hemothorax, pneumothorax, or identifiable lung injury.

Approximately 34 minutes after the injury, the patient arrived at the trauma center. He was awake, alert, and hemodynamically stable with tachycardia up to 115 beats per minute. The impaled object protruded approximately 50cm anteriorly and 28cm posteriorly through the right upper chest wall (Figure 1). A chest X-ray taken in the trauma bay showed the impalement object and no identifiable acute pulmonary injury (Figure 2).

The patient was taken directly to the operating room. He was intubated with a single-lumen endotracheal tube and placed in the left lateral decubitus position. We began with a local wound exploration, by making an incision at the anterior right upper chest, extending the wound 12 cm medially and 8 cm laterally. Glass and debris were removed from the wound. We identified the subclavian artery, subclavian vein, and brachial plexus medially and the axillary artery, axillary vein, and brachial plexus branches laterally. The metal fence post had impaled below his clavicle and above his pleural cavity, 2mm cephalad to the major neurovascular structures. There was a small 0.5mm arteriotomy with mild bleeding from the subclavian artery proximal to the injury that was primarily repaired with a nonabsorbable suture. Oozing was noted from the tributaries of the pectoralis major, which were suture ligated.

We then made an incision to extend the posterior right upper thorax wound 4cm both medially and laterally. Vascular control was obtained through the anterior incision by clamping the subclavian artery and subclavian vein proximally, and the axillary artery and axillary vein distally. The impalement object was removed without complication. The anterior and posterior wounds were irrigated thoroughly with normal saline, and nonviable and devitalized soft tissue was resected. The pectoralis major muscle anteriorly and the rhomboid and latissimus dorsi muscles posteriorly were reapproximated, and the surgical site was closed with staples. A right-sided 28 French chest tube was placed.

The patient was kept intubated and transferred to the intensive care unit. Postoperatively, computed tomography revealed comminuted fractures of the right scapula and right clavicle. The patient was extubated on postoperative day one. He received tetanus prophylaxis and antibiotic coverage with cefazolin for three days. Orthopedic surgery deferred surgical fixation due to wound contamination. His hospital course was complicated by a right-sided pneumothorax, which resolved, and the patient was discharged home on postoperative day 11. Three months after his injury, his right upper thorax incisions were healing, and he had a full range of motion of his right upper extremity, with mild limitation with abduction.

## Discussion

Impalement injuries can be classified as type 1 or type 2, and simple or complex. A type 1 impalement injury occurs when a moving body falls onto an impaling object, and type 2 occurs when the moving object impales a stationary human body, such as in road traffic accidents [4]. A simple impalement occurs with objects such as knives or arrows. A complex impalement involves a transfixed impaled object that traps or pins the patient in an inescapable position [5]. The injury described in our report is a complex type 2 impalement.

Right-sided thoracic impalements are more common than left, presumably due to the reduced risk of a penetrating injury to the heart or major vessels which lie predominately within the left thorax, such as the aorta [6]. Patients who survive transport to a trauma center have most likely avoided significant injury to the cardiovascular system, and organ injury would be isolated to the lung [6,7].

Although the management of impalement injuries depends entirely on the anecdote, the suggested systematic approach (Table 1) can improve patient outcomes [6-11]. An expedient extrication with minimal manipulation of the impaled foreign object will avoid loss of the tamponade effect [8-10]. Literature discourages the use of preoperative imaging; however, depending on the site of injury, imaging such as a chest X-ray, computed tomography, or aortic angiography can guide surgical management [8]. In our patient, an X-ray in the trauma bay confirmed no acute pulmonary pathology, changing the surgical approach from a right thoracotomy to a local wound exploration. Minimal manipulation of the impaled object should occur prior to operative intervention [12].

**Table 1:** General guidelines for the management of thoracoabdominal impalement injuries, which can be applied to each patient and modified to the location and mechanism of injury.

Table 1. Guidelines for the Management of Thoracoabdominal Impalement	
1.	Cautious extrication from scene with minimal manipulation of penetrating object.
2.	Rapid transport from scene to trauma center.
3.	Leave object in situ to maintain tamponade of vessels and structures.
4.	Obtain wide operative exposure.
5.	Ensure major vessel control before object removal.
6.	Resect necrotic tissue, but preserve lung tissue as possible to prevent empyema.
7.	Contaminated wounds require lavage, debridement, antibiotics, and tetanus prophylaxis.
8.	Close the chest when situation permits; reconstructive surgery may be necessary.

Operative principles of impalement injuries must include wide exposure, vascular control, organ and tissue repair, and removal of the foreign body [13]. Preoperative planning is vital, and unconventional incisions and positioning may be required to obtain optimal exposure [7,8]. The decubitus lateralis position is the ideal choice, but to achieve this, the impaled object may need to be cut more posteriorly. Dual-lumen intubation can be helpful to provide single-lung ventilation, to better visualize and repair a lung injury [14].

When possible, proximal and distal vascular control is mandatory prior to the removal of the impaled object to prevent significant blood loss [10,11]. In the case of our patient, we extended his anterior right upper chest wound both medially and laterally along the course of the major vascular structures. If vascular control was unsuccessful, then a midline sternotomy was indicated to access the proximal subclavian artery and vein. All debris and necrotic tissue should be removed from the wound to decrease the risk of sepsis [10]. If the impalement causes direct injury to the lung, then all viable lung should be preserved to prevent dead space and minimize the risk of postoperative empyema [10]. If possible, closure of the wound should be attempted; however, given the complexity of impalement wounds, a delayed plastic reconstruction may be necessary [7,10]. Impalement wounds are typically grossly contaminated with soil pathogens, requiring aggressive irrigation, debridement, tetanus prophylaxis, and antibiotic coverage [9].

## Conclusions

Impalement injuries are rare and require a collaborative effort between emergency responders and the trauma team. A systematic approach that leads to improved patient outcomes should include minimal handling of the impaled object, expedient transfer to a level-one trauma center, emergent operative intervention, vascular control prior to removal of the foreign object, and aggressive irrigation and debridement of the wound.
